# The E3 ubiquitin ligase HERC1 controls the ERK signaling pathway targeting C-RAF for degradation

**DOI:** 10.18632/oncotarget.25847

**Published:** 2018-07-31

**Authors:** Taiane Schneider, Arturo Martinez-Martinez, Monica Cubillos-Rojas, Ramon Bartrons, Francesc Ventura, Jose Luis Rosa

**Affiliations:** ^1^ Departament de Ciències Fisiològiques, IDIBELL, Campus Bellvitge, Universitat de Barcelona, L’Hospitalet de Llobregat, Barcelona, Spain

**Keywords:** ubiquitin, ERK, RAF, proliferation, protein degradation

## Abstract

The RAF/MEK/ERK cascade is a conserved intracellular signaling pathway that controls fundamental cellular processes including growth, proliferation, differentiation, survival and migration. Aberrant regulation of this signaling pathway has long been associated with human cancers. A major point of regulation of this pathway occurs at the level of the serine/threonine protein kinase C-RAF. Here, we show how the E3 ubiquitin ligase HERC1 regulates ERK signaling. HERC1 knockdown induced cellular proliferation, which is associated with an increase in ERK phosphorylation and in C-RAF protein levels. We demonstrate that overexpression of wild-type C-RAF is sufficient to increase ERK phosphorylation. Experiments with pharmacological inhibitors of RAF activity, or with interference RNA, show that the regulation of ERK phosphorylation by HERC1 is RAF-dependent. Immunoprecipitation, pull-down and confocal fluorescence microscopy experiments demonstrate an interaction between HERC1 and C-RAF proteins. Mechanistically, HERC1 controls C-RAF stability by regulating its polyubiquitylation in a lysine 48-linked chain. *In vitro* ubiquitylation assays indicate that C-RAF is a substrate of the E3 ubiquitin ligase HERC1. Altogether, we show how HERC1 can regulate cell proliferation through the activation of ERK signaling by a mechanism that affects C-RAF’s stability.

## INTRODUCTION

The RAF/MEK/ERK signaling pathway controls many fundamental cellular processes including growth, proliferation, differentiation, survival and migration. In this conserved intracellular pathway, upon the stimulation of receptor tyrosine kinases at the cell surface, a guanine nucleotide exchange factor promotes the activation of GTPase RAS through the exchange of GDP to GTP. GTP-bound RAS interacts and activates RAF at the plasma membrane. Subsequently, activated RAF phosphorylates and activates MEK, which in turn phosphorylates and activates ERK. ERK phosphorylates hundreds of substrates, which mediate many of the pleiotropic effects of this pathway. Dysregulation of this phosphorylation cascade has long been associated with human cancers. Thus, ERK activation is observed in most human cancers and, in about one-third of them, it is driven by mutational activation of its pathway components [1–3]

Like protein phosphorylation, protein ubiquitylation has also emerged as a prevalent posttranslational modification utilized in the regulation of cellular processes. It consists of the covalent attachment of a small polypeptide ubiquitin to target proteins. This intracellular process occurs in a three-step reaction requiring three different enzymes: a ubiquitin-activating enzyme (E1), a ubiquitin conjugating enzyme (E2), and a ubiquitin ligase enzyme (E3). Ubiquitin is first activated by an E1, which then promotes the conjugation of ubiquitin onto an E2. Finally, ubiquitin is conjugated to a lysine residue of a target protein by an E3, which acts as a specific adaptor for the target protein. This cycle of ubiquitin conjugation can be repeated in order to build different ubiquitin chains with different functions. [4–7].

The HERC family proteins are E3 ubiquitin ligases. They contain two characteristic domains: the HECT domain, and one or more RCC1-like domains. Proteins containing RCC1-like domains function as GTPase regulators, whereas proteins with HECT domains function as E3 ubiquitin ligases [8]. These two activities are essential in many important cellular processes such as cell cycle, cell signaling, and membrane trafficking. Mutations affecting these domains have been mainly associated with cancer, neurological disorders, male fertility, and the antiviral response [9, 10]. In humans, six HERC genes have been reported which encode two subgroups of HERC proteins: large (HERC1-2) and small (HERC3-6). The giant HERC1 protein was the first to be identified. It is expressed ubiquitously in mammalian tissues, with slightly higher levels in brain and testis, and the lowest levels in liver. Its subcellular localization is restricted to the cytoplasm and Golgi/vesicular-like membrane compartments [11]. HERC1 has been implicated in membrane trafficking, cell proliferation/growth and apoptosis through its interaction with ARF, Rab, Clathrin, M2-pyruvate kinase, TSC2 and BAK proteins [11–15]. *In vivo* studies have implicated HERC1 in neurological disorders. Evidence was first obtained with the *tambaleante* mutant mouse, which carries a spontaneous Gly483Glu mutation in the amino-terminal RCC1-like domain of the HERC1 protein and causes an increase of mutated protein levels. These animals are smaller, have a reduced lifespan, and an ataxic syndrome caused by the almost complete loss of cerebellar Purkinje cells during adult life [16]. More recent analyses have also identified alterations at neuromuscular junctions in these mice, as well as in other neurons [17–19]. Biallelic mutations in human HERC1 have been associated with overgrowth, intellectual disability and some autistic features [20–24]. For these reasons, it has been recommended to consider HERC1 mutations in the differential diagnosis of severe intellectual disability and behavioral problems [23, 24]. HERC1 has also been involved in cancer. Mutations in HERC1 were detected in leukemias [25–27], breast cancers [28, 29] and, more recently, HERC1 has been associated with non-melanoma skin cancer through regulation of E6-mediated BAK degradation [15].

Despite previous evidence indicating a role of E3 ubiquitin ligase HERC1 in proliferative processes, there is very little information on the molecular mechanisms involved, as well as on the ubiquitylation substrates of this ligase. In this study, we found that cell proliferation was increased by ERK activation in a HERC1-dependent manner; HERC1 interacts with C-RAF and regulates its stability. Our data show that the E3 ubiquitin ligase HERC1 controls the ERK signaling pathway by regulation of C-RAF protein levels via ubiquitylation.

## RESULTS

### HERC1 regulates cell proliferation and ERK signaling

**Figure 1:**
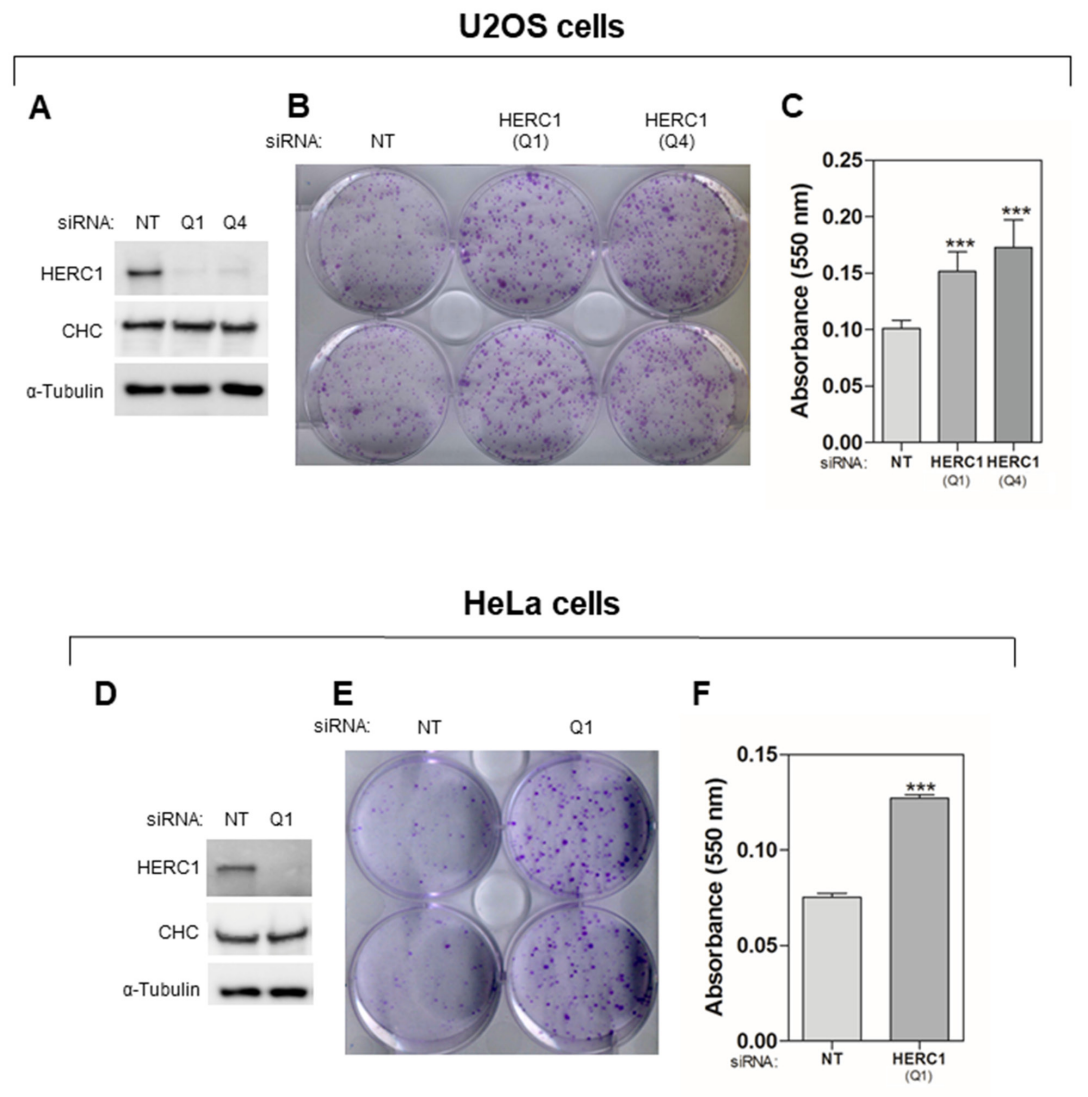
HERC1 regulates cell proliferation Cells were transfected with non-targeting (NT) or HERC1 siRNA. For U2OS cells, two different HERC1 siRNAs (Q1 and Q4) were used. **(A, D)** Seventy-two hours later, lysates were analyzed by immunoblotting with antibodies against the indicated proteins. **(B, C, E, F)** Colony formation assays were performed as indicated in “Materials and Methods”. After 12-15 days, cells were stained with crystal violet (B, E). Crystal violet absorbance at 550 nm was quantified (n = 6 for U2OS cells; n = 3 for HeLa cells) (C, F). CHC: Clathrin heavy chain. Data are expressed as mean ± S.E.M. Statistical analysis was carried out as described under “Materials and Methods”. The differences are shown with respect to NT siRNA. ^***^, p< 0.001.

Previous work suggests an important role of the E3 ubiquitin ligase HERC1 in cancer [15, 25–29]. In order to study the function of HERC1 in cell proliferation, we decided to perform a clonogenic assay in HERC1-depleted human cancer cells. Human osteosarcoma U2OS cells, frequently used in cell signaling studies, were transfected with two different small interfering RNA (siRNA) of HERC1 (Q1 or Q4). Non-targeting (NT) siRNA was also transfected and used as a negative control. A great decrease in HERC1 protein levels was observed by Western blot analysis 72 hours after transfection (Figure 1A). HERC1 depletion had no effect on different loading controls such as α-Tubulin and Clathrin Heavy Chain (CHC) (Figure 1). Transfected cells were grown for 12-15 days in a clonogenic assay as indicated in “Materials and Methods”. After this time, cells were visualized by crystal violet staining (Figure 1B). A significant increase in the number of colonies was observed in cells transfected with HERC1 siRNA when compared with cells transfected with NT siRNA. These observations were quantified by an absorbance analysis of the crystal violet staining (Figure 1C). To check whether this effect could be observed in other cell types, cervical cancer HeLa cells were also transfected with the above siRNAs. We observed similar effects in this human cell line (Figure 1D-F) as in U2OS cells (Figure 1A-C). These results suggest a regulatory role of HERC1 in cell proliferation.

**Figure 2:**
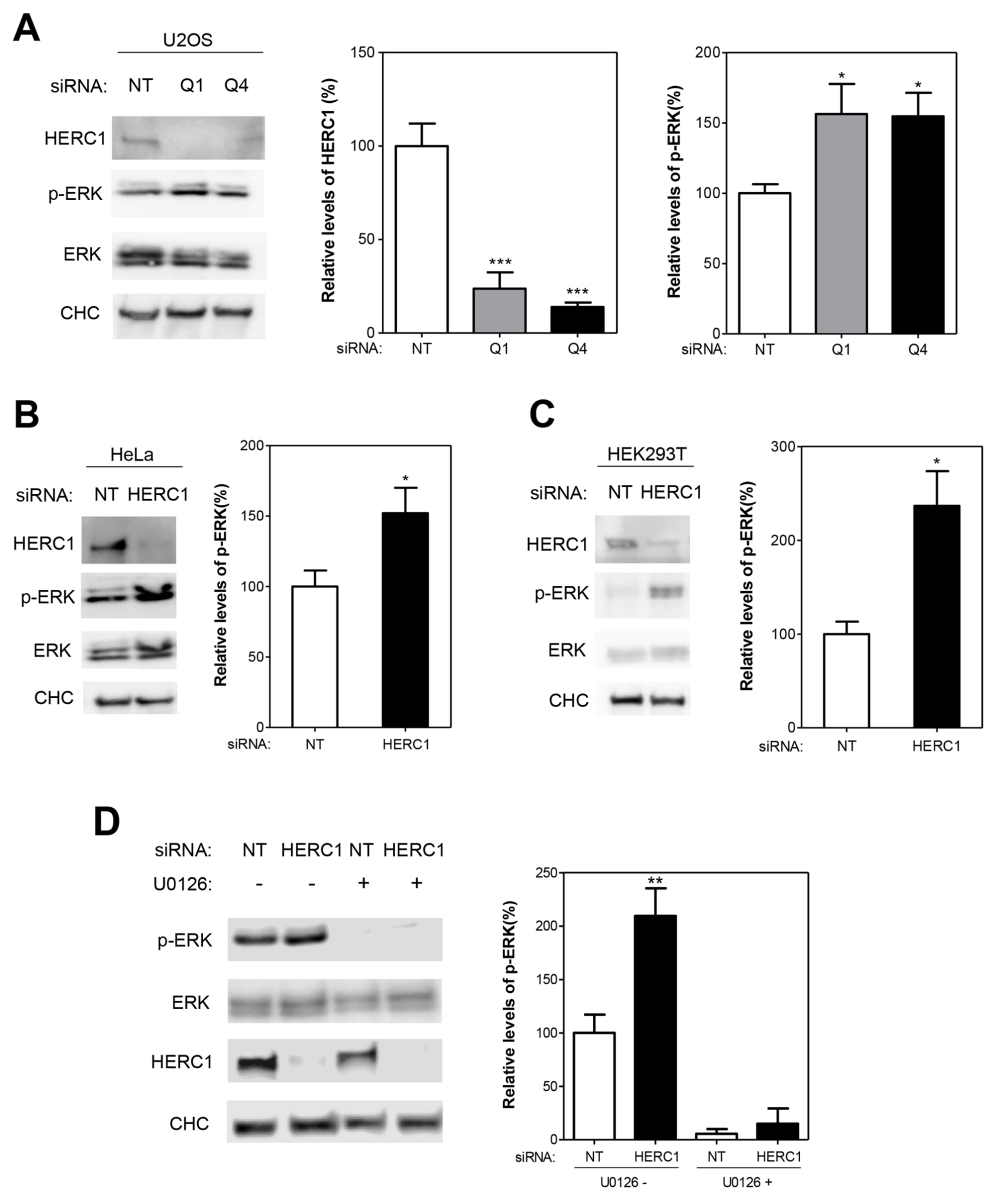
HERC1 regulates ERK signaling **(A)** Lysates from U2OS cells transfected with NT or HERC1 siRNAs (Q1 or Q4) were analyzed by immunoblotting with antibodies against the indicated proteins. Levels of HERC1 and p-ERK were quantified and normalized with respect to CHC and ERK levels, respectively (n = 4). **(B-C)** Lysates from HeLa or HEK-293T cells were analyzed as above (n = 6 for HeLa cells; n = 4 for HEK-293T cells). **(D)** U2OS cells were transfected with NT or HERC1 (Q1) siRNAs. Seventy-two hours later, cells were treated with the inhibitor U0126 (10 μM) for 1 hour. Lysates were analyzed as in (A) (n = 3). Data are expressed as mean ± S.E.M. Statistical analysis was carried out as described under “Materials and Methods”. The differences are shown with respect to NT siRNA. ^*^, p< 0.05; ^**^, p< 0.01; ^***^, p< 0.001.

Since ERK activation is required for cell proliferation, the activation of cell proliferation by HERC1 knockdown (Figure 1) led us to analyze whether ERK signaling was activated under these conditions. The analysis of ERK1/2 phosphorylation with phospho-specific antibodies is frequently used to assess ERK activation. We observed a significant increase of ERK1/2 phosphorylation (p-ERK) in U2OS cells transfected with HERC1 siRNA (Figure 2A). The increase in p-ERK cannot be due to an augmented level of ERK proteins because HERC1 depletion does not modify ERK protein levels (Figure 2A). Similar results were observed in HeLa cells and in Human embryonic kidney cells 293T (HEK-293T) (Figure 2B-C). ERK is phosphorylated by the ERK kinase MEK. To show that the increase of p-ERK protein levels was due to an increase in MEK activity, we used a highly selective inhibitor of MEK activity: U0126. Treatment with this inhibitor greatly reduced the ability of HERC1 siRNA to induce ERK phosphorylation (Figure 2D). Although we cannot discard that other factors could be involved in the regulation of ERK phosphorylation, these data show a regulation of ERK signaling by HERC1.

### HERC1 regulates ERK pathway by RAF activation

**Figure 3:**
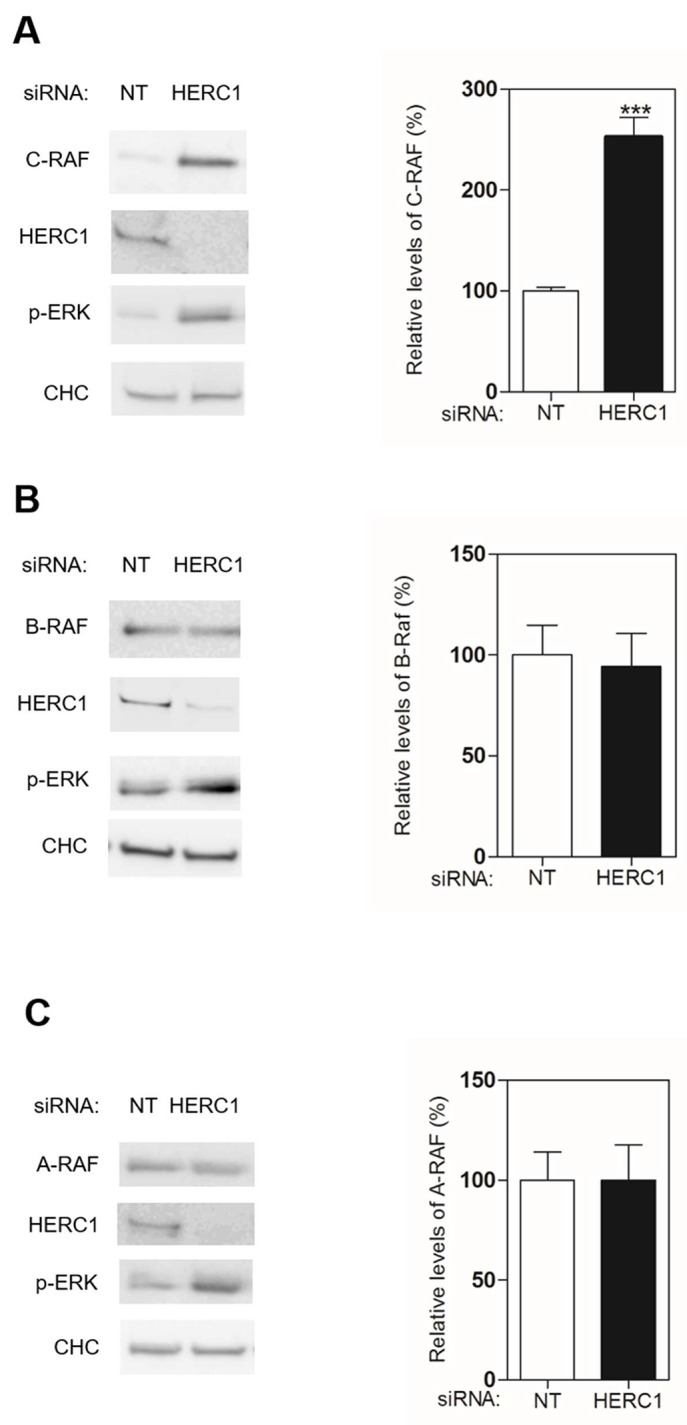
HERC1 regulates C-RAF protein levels **(A-C)** Lysates from U2OS cells transfected with NT or HERC1 siRNAs were analyzed by immunoblotting with antibodies against the indicated proteins. Levels of RAF proteins were quantified and normalized with respect to CHC levels (n = 4). Data are expressed as mean ± S.E.M. Statistical analysis was carried out as described under “Materials and Methods”. The differences are shown with respect to NT siRNA. ^***^, p< 0.001.

**Figure 4:**
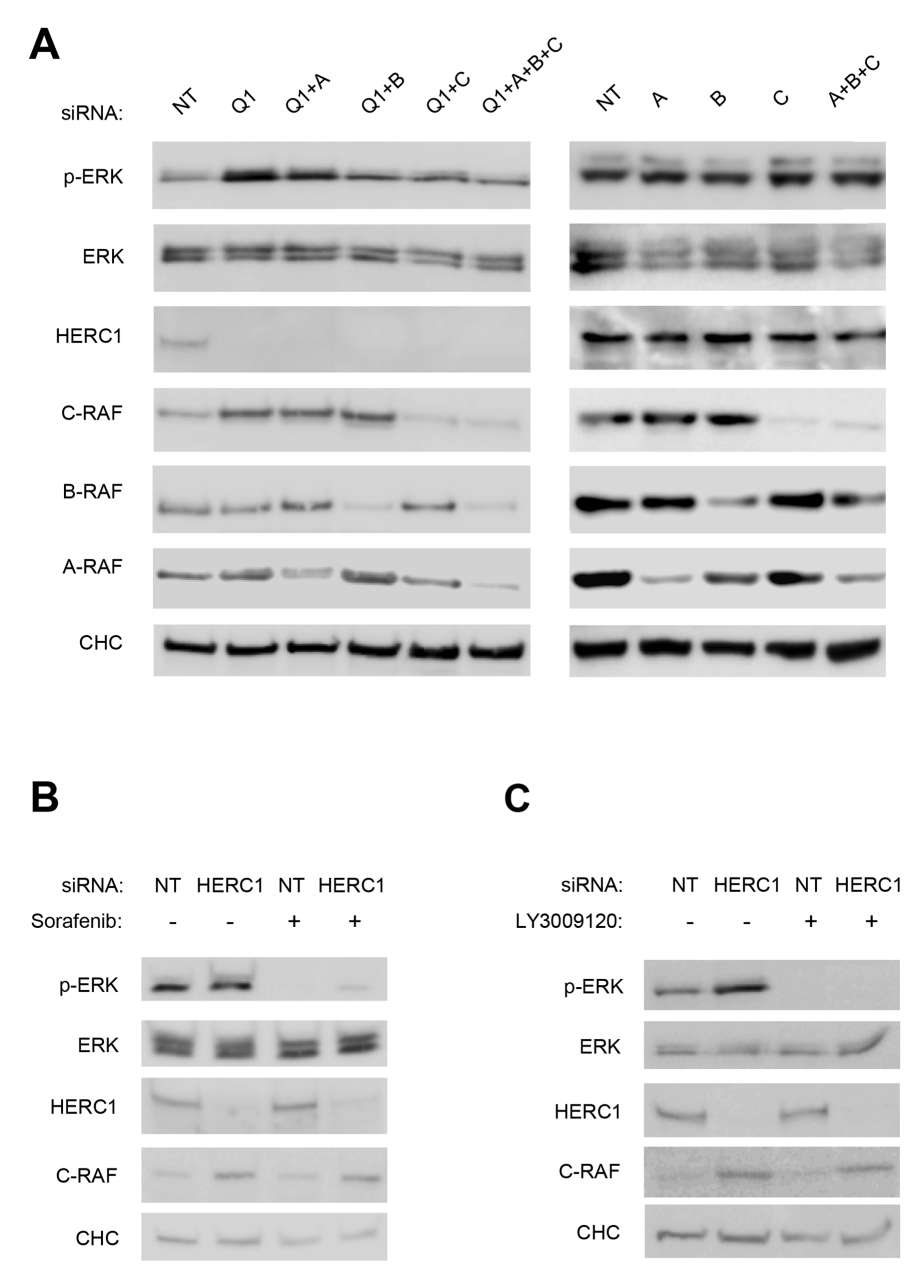
ERK activation is RAF dependent **(A)** Lysates from U2OS cells transfected with the indicated siRNA (NT: non-targeting, Q1: HERC1, A: A-RAF, B: B-RAF, and C: C-RAF) were analyzed by immunoblotting with antibodies against the indicated proteins. **(B, C)** U2OS cells were transfected with NT or HERC1 siRNAs. Seventy-two hours later, cells were treated with 10 μM Sorafenib or 10 μM LY3009120 for 2 hours. Lysates were analyzed as in (A). Data are representative of at least three independent experiments.

Activation of the ERK signaling pathway occurs upon subsequent and sequential activation of RAF, MEK, and ERK kinases. We investigated the involvement of the first kinase of this signal transduction cascade in the activation of ERK by HERC1 knockdown. Since there are three isoenzymes in mammals, we analyzed protein levels of A-RAF, B-RAF and C-RAF by immunoblotting in U2OS cells transfected with siRNA of HERC1. NT siRNA was used as a negative control. We observed in conditions where HERC1 knockdown activated ERK (p-ERK), that the level of C-RAF protein increased (Figure 3A). In the same conditions, A-RAF and B-RAF levels were not significantly modified (Figure 3B-C). This result would suggest that the increase of C-RAF protein level could be responsible for ERK activation by HERC1 knockdown. To confirm this point, we repeated these experiments by transfecting U2OS cells with C-RAF siRNA. C-RAF depletion decreased ERK activation produced by the HERC1 knockdown (Figure 4A, compare lanes 5 and 2). However, we also observed a remnant level of p-ERK slightly higher than in NT siRNA cells (Figure 4A, compare lanes 5 and 1). We analyzed A-RAF and B-RAF proteins in order to test the possibility of a compensatory mechanism being contributed by other RAF isoforms [1, 30]. We did not observe significant changes in the amount of these proteins in the presence of C-RAF siRNA (Figure 4A, lane 5). We also performed HERC1 knockdown experiments with A-RAF siRNA, B-RAF siRNA, and with siRNA of the three isoforms. Under these conditions, B-RAF depletion decreased ERK activation produced by HERC1 knockdown to a similar extent as C-RAF siRNA (Figure 4A, lane 4), suggesting the involvement of C-RAF/B-RAF heterodimers in this signaling. A-RAF depletion only slightly decreased ERK activation produced by HERC1 knockdown (Figure 4A, compare lanes 3 and 2), suggesting that A-RAF contributes less in this type of signaling. In agreement with all these data, knockdown of the three isoforms did not result in ERK activation (Figure 4A, lane 6).

To confirm that ERK activation by HERC1 knockdown was dependent on RAF activity, we used two different inhibitors of RAF activity: Sorafenib and LY3009120. We observed that the treatment of U2OS cells with these inhibitors impaired ERK activation from HERC1 knockdown (Figure 4B-C). Interestingly, a small increase of p-ERK after HERC1 knockdown was observed in the presence of Soranefib (Figure 4B, lane 4), but not in the presence of LY3009120 (Figure 4C, lane 4). This could be due to the different specificities of these RAF inhibitors: Soranefib is a specific inhibitor of B-RAF and C-RAF, whereas LY3009120 is a pan-RAF inhibitor that inhibits all RAF isoforms.

**Figure 5:**
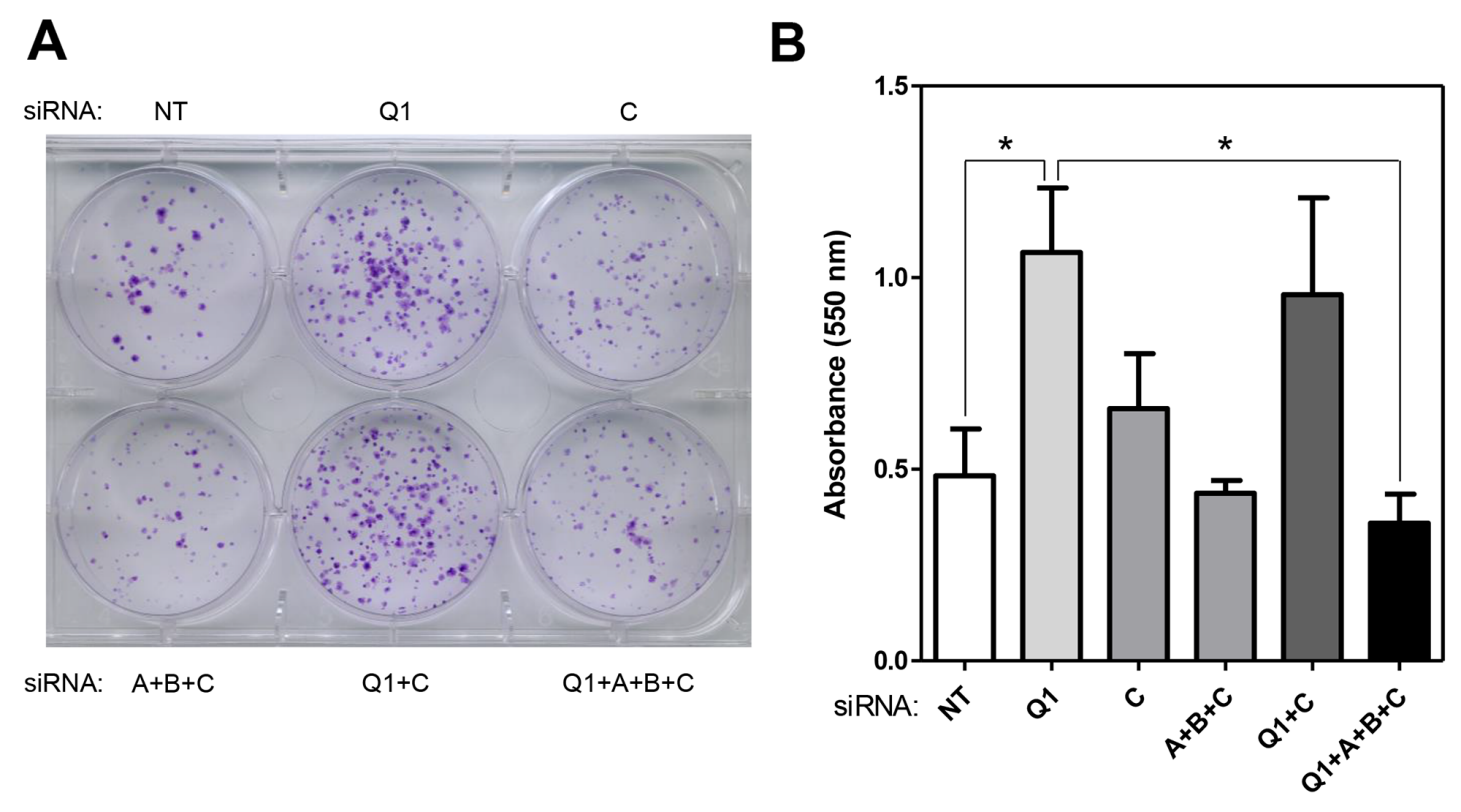
Regulation of cell proliferation by HERC1 is RAF dependent HeLa cells were transfected with the indicated siRNA (NT: non-targeting, Q1: HERC1, A: A-RAF, B: B-RAF, and C: C-RAF). **(A)** Colony formation assay was performed as indicated in “Materials and Methods” and cells were stained with crystal violet. **(B)** Crystal violet absorbance was quantified (n = 3). Data are expressed as mean ± S.E.M. Statistical analysis was carried out as described under “Materials and Methods”. ^*^, p< 0.05.

To conclude that HERC1 knockdown promotes cell proliferation (Figure 1) via C-RAF stabilization (Figure 3) and RAF activation (Figure 4), cell proliferation must be reverted by C-RAF inhibition or RAF knockdown. We observed in conditions where HERC1 knockdown increased the number of colonies, that it was necessary to knockdown the three RAF isoforms in order to avoid this increase (Figure 5).

### Overexpression of C-RAF is enough to activate C-RAF and ERK signaling

**Figure 6:**
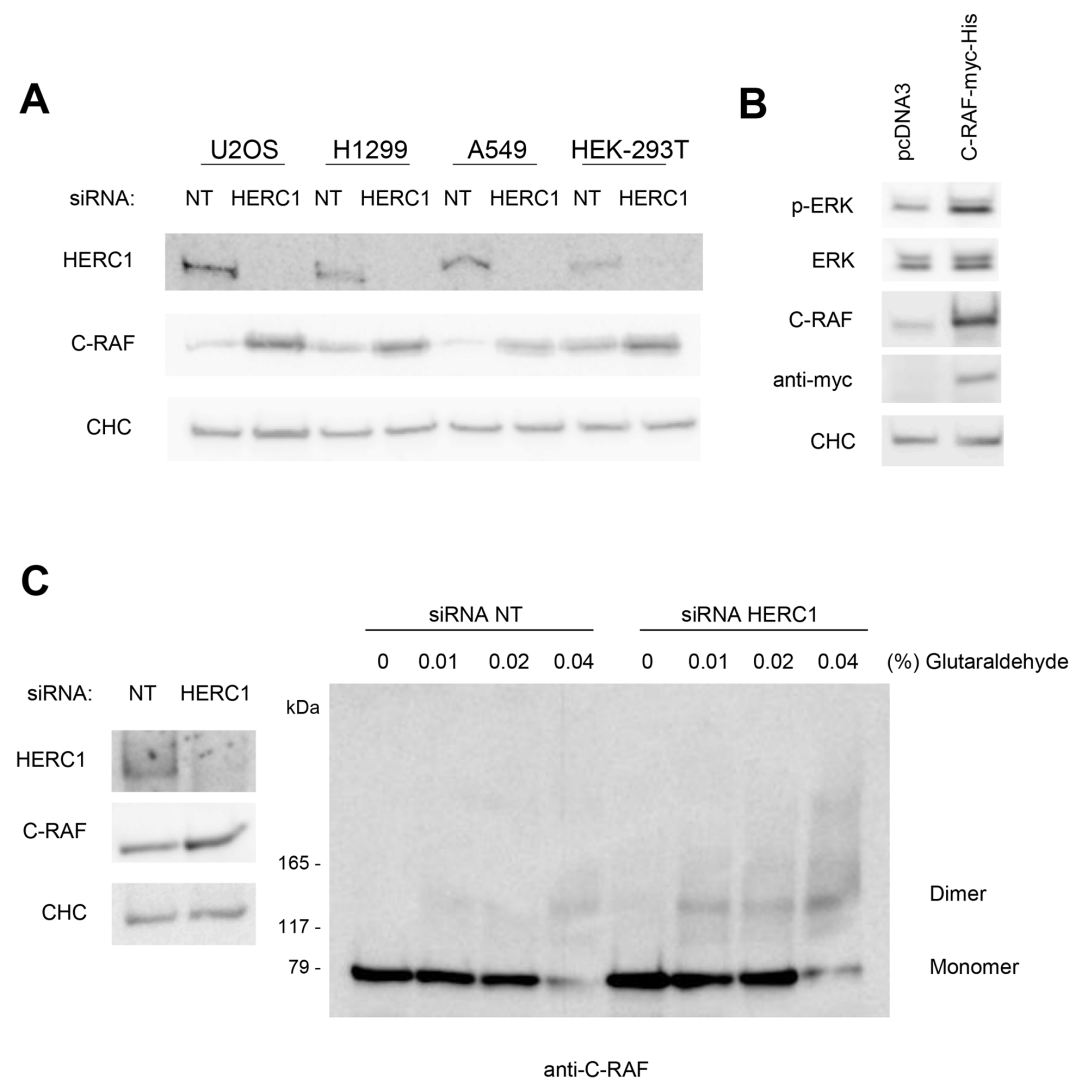
The increase of C-RAF activates ERK signaling **(A)** Cells were transfected with NT or HERC1 siRNAs. Lysates were analyzed by immunoblotting with antibodies against the indicated proteins. CHC protein was analyzed as a loading control. 293T: HEK-293T. **(B)** U2OS cells were transfected with the indicated plasmids. Forty-eight hours later, lysates were analyzed as in (A). **(C)** Lysates from U2OS cells transfected with NT or HERC1 siRNAs were incubated on ice with glutaraldehyde at the indicated concentrations for 30 min, and C-RAF oligomerization was analyzed by immunoblotting with anti-C-RAF antibody as indicated under “Materials and Methods”. Data are representative of at least three independent experiments.

The increase in C-RAF protein by HERC1 knockdown (Figure 3 and 4) indicates that HERC1 could be involved in the regulation of C-RAF stability. We confirmed this regulation in additional human cancer cells (H1299 and A549). This regulation was independent of p53 expression (H1299 cells do not express the p53 protein and A549 cells express wild-type p53) (Figure 6A). To be sure that this regulation was not restricted to human cancer cells, we also demonstrated this increase in C-RAF protein amount by HERC1 knockdown in HEK-293T cells (Figure 6A). To show that the increase of C-RAF is enough to activate ERK, U2OS cells were transfected with a plasmid expressing wild-type C-RAF. We observed how C-RAF overexpression produced an increase in ERK phosphorylation (Figure 6B). These data show that an increase of the C-RAF quantity is enough to activate ERK signaling.

C-RAF activation requires C-RAF oligomerization [1, 31, 32]. To show that C-RAF was active after HERC1 knockdown, we analyzed C-RAF oligomerization using a protein cross-linking assay [33]. U2OS cells were transfected with HERC1 or NT siRNA and analyzed 72 hours later. Cell lysates were isolated, treated with increasing amounts of glutaraldehyde, and analyzed by SDS-PAGE and immunoblotting with anti-C-RAF antibodies. As shown in Figure 6C, C-RAF oligomerization was increased in HERC1 knockdown cells.

### HERC1 interacts with C-RAF

**Figure 7:**
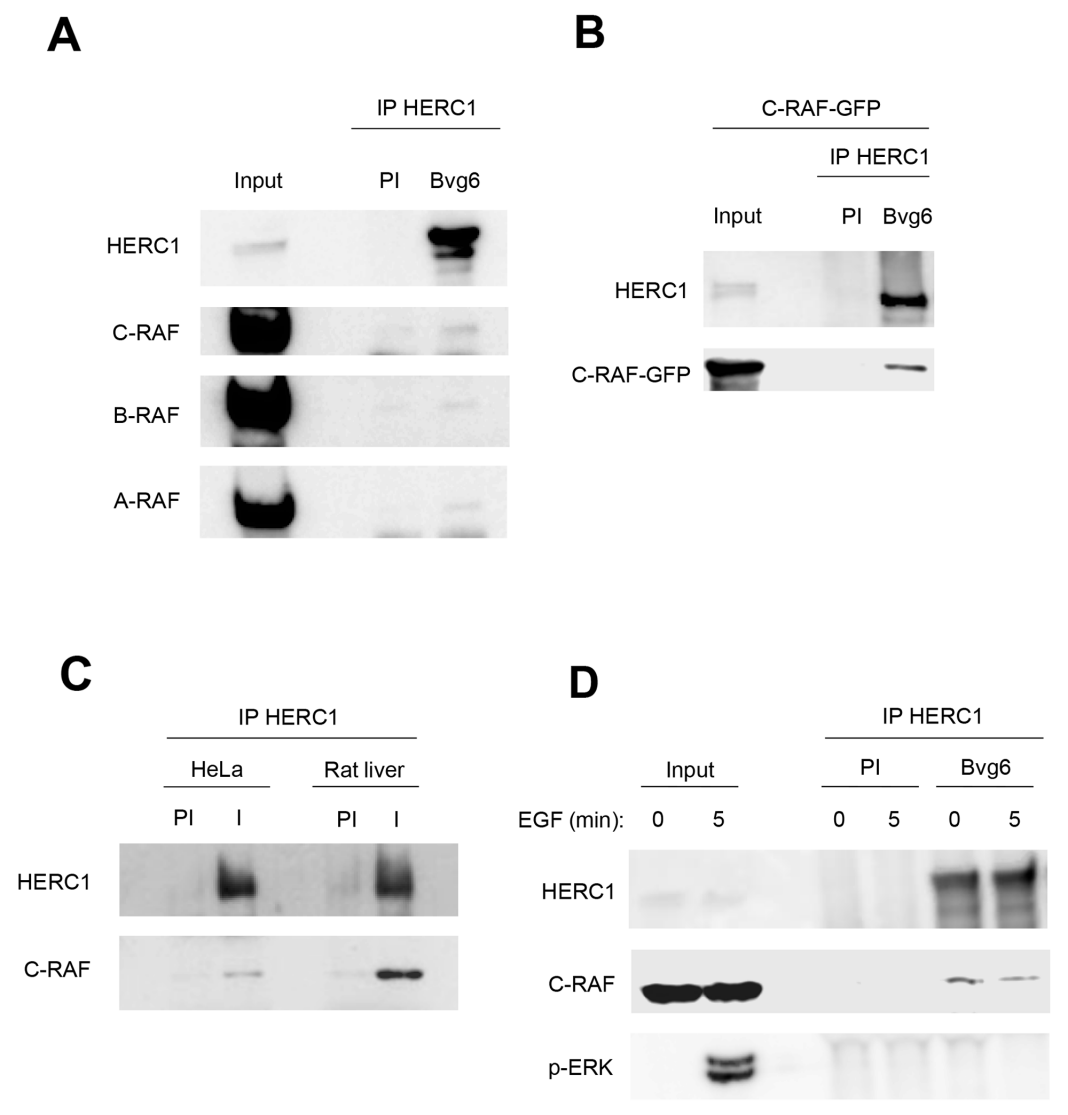
HERC1 interacts with C-RAF **(A)** Supernatants (input) of lysates from HEK-293T cells were immunoprecipitated (IP) using anti-HERC1 antibodies (I) and analyzed by immunoblotting with antibodies against the indicated proteins. Pre-immune serum (PI) was used as a negative control. **(B)** Similar experiments were performed in HEK-293T cells transfected with C-RAF-GFP, and **(C)** in HeLa cells and in rat liver. **(D)** HEK-293T cells were deprived of serum overnight and stimulated with 120 ng/ml EGF for 5 min. Lysates were treated as in (A). Data are representative of at least three independent experiments.

To analyze whether HERC1 can interact with C-RAF, we performed immunoprecipitation experiments in HEK-293T cells with a specific antibody (Bvg6) against HERC1. This antibody could coimmunoprecipitate endogenous C-RAF with endogenous HERC1 (Figure 7A). Pre-immune serum (PI) was used as a negative control. In agreement with the formation of oligomers between RAF proteins [34–36], A-RAF and B-RAF isoforms were also present in the coimmunoprecipitate (Figure 7A). The interaction between HERC1 and C-RAF proteins was more evident in HEK-293T cells transfected with C-RAF-GFP (Figure 7B). The interaction between endogenous proteins was also confirmed in HeLa cells and in rat liver (Figure 7C).

We analyzed whether activation of ERK signaling with epidermal growth factor (EGF) could modulate this interaction. HEK-293T cells were deprived of serum overnight and then stimulated with EGF for 5 min. An increase of p-ERK was detected (Figure 7D). Under these conditions, we did not observe a significant change in the interaction between HERC1 and C-RAF proteins.

**Figure 8:**
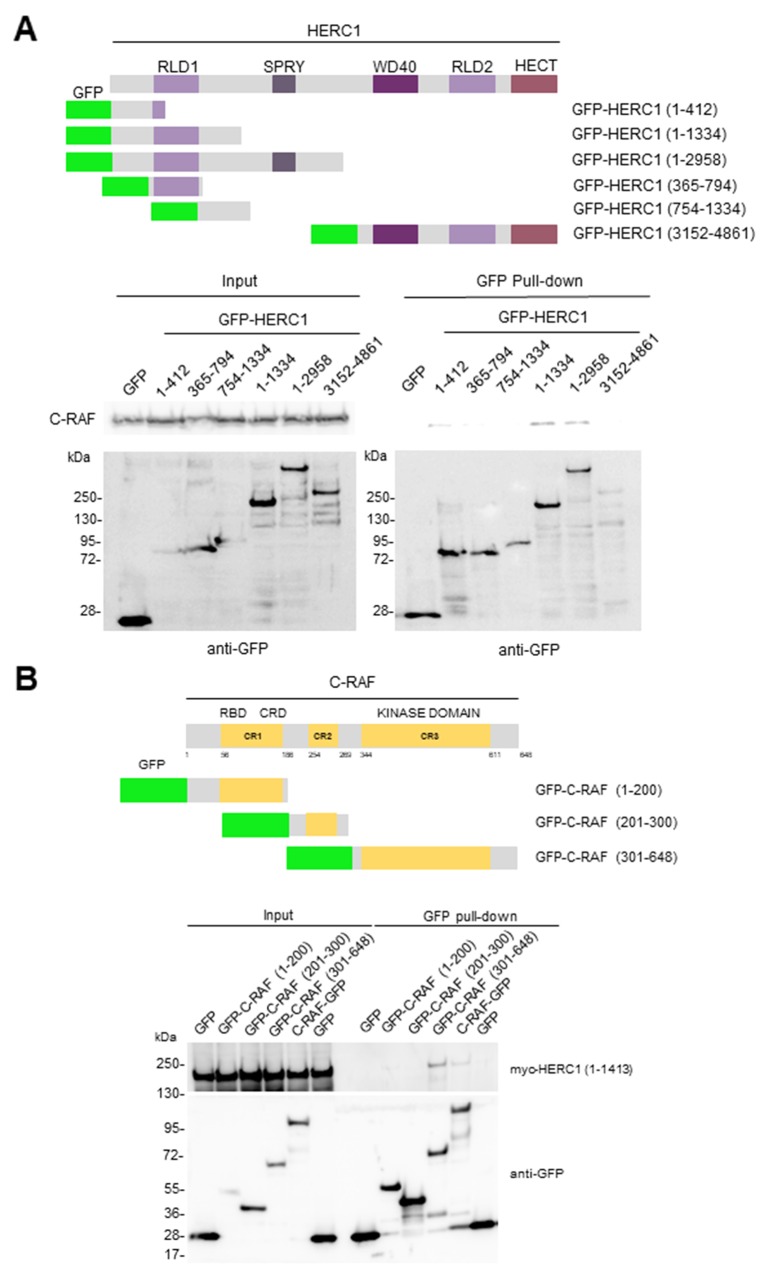
Domains involved in the interaction between HERC1 and C-RAF proteins Structure of HERC1 **(A)** and C-RAF **(B)** proteins. Relevant domains are indicated. GFP fusion proteins with the amino acid residues expressed are shown. Pull-down experiments were performed in HEK-293T cells transfected with GFP or GFP-HERC1 fusion constructs (A), or with myc-HERC1 (1-1413) and GFP or GFP-C-RAF fusion constructs (B). About 24-48 hours post-transfection, lysates from these cells were incubated with GFP-binding beads as indicated in “Materials and Methods”. Proteins retained in the resin were analyzed by immunoblotting with antibodies against the indicated proteins.

**Figure 9:**
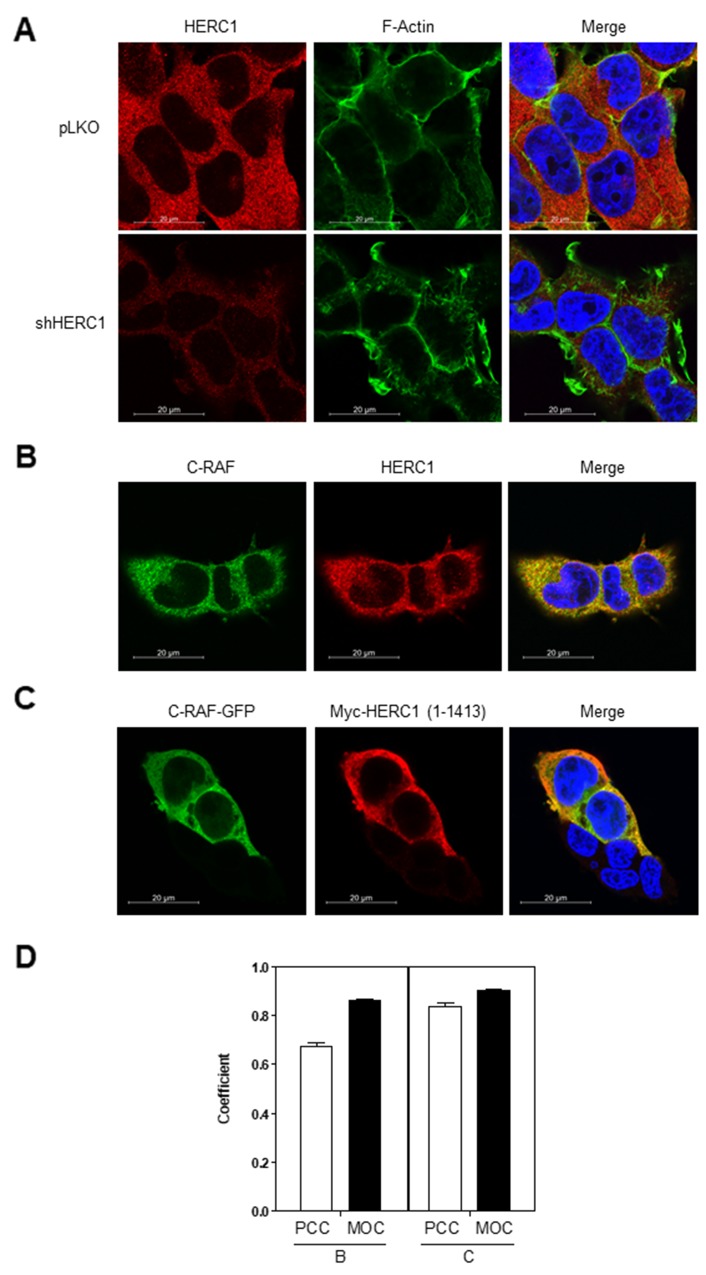
Colocalization of HERC1 and C-RAF proteins **(A)** Specificity of the anti-HERC1 antibody. HEK-293T cells infected with lentivirus (shHERC1: shRNA of HERC1; pLKO: the lentivirus plasmid vector as negative control) were stained for HERC1 (red), F-actin (green) and nuclei (blue), and analyzed by confocal microscopy. **(B)** Confocal immunofluorescence analysis shows partial colocalization of endogenous HERC1 and C-RAF proteins. HEK-293T cells were stained for HERC1 (red), C-RAF (green) and nuclei (blue), and analyzed by confocal microscopy. Colocalization is observed in the merge panel. **(C)** HEK-293T cells transfected with C-RAF-GFP and myc-HERC1 (1-1413) constructs were stained for myc (myc-HERC1) (red), GFP (C-RAF-GFP) (green) and nuclei (blue), and analyzed by confocal microscopy. Colocalization is observed in the merge panel. **(D)** Pearson’s correlation coefficient (POC) and the Manders’ overlap coefficient (MOC) are showed (56 cells were used for colocalization analysis of endogenous proteins (B) and 19 cells for analysis of transfected constructs (C)). Data are representative of three independent experiments.

To identify the region of HERC1 interacting with C-RAF, we expressed a series of GFP-HERC1 fusion proteins (Figure 8A) in HEK-293T cells, and performed pull-down assays with GFP-binding beads. Constructs GFP-HERC1 (1-412), GFP-HERC1 (1-1334) and GFP-HERC1 (1-2958) could pull-down endogenous C-RAF protein, indicating that amino acid residues 1-412 of HERC1 are enough to interact with C-RAF. To map the region of C-RAF involved in the interaction with HERC1, we transfected HEK-293T cells with plasmids expressing myc-HERC1 (amino acid residues 1-1413) and several GFP-C-RAF fusion proteins (Figure 8B). Pull-down experiments with GFP-binding beads showed that amino acid residues 301-648 of C-RAF, which encloses its kinase domain, are involved in this interaction. Additionally, confocal immunofluorescence analysis showed the colocalization of endogenous C-RAF and HERC1 proteins, and overexpressed C-RAF-GFP and myc-HERC1 (1-1413) proteins (Figure 9). Altogether, these data confirm the interaction between HERC1 and C-RAF proteins.

### The ubiquitin ligase HERC1 regulates C-RAF ubiquitylation

**Figure 10:**
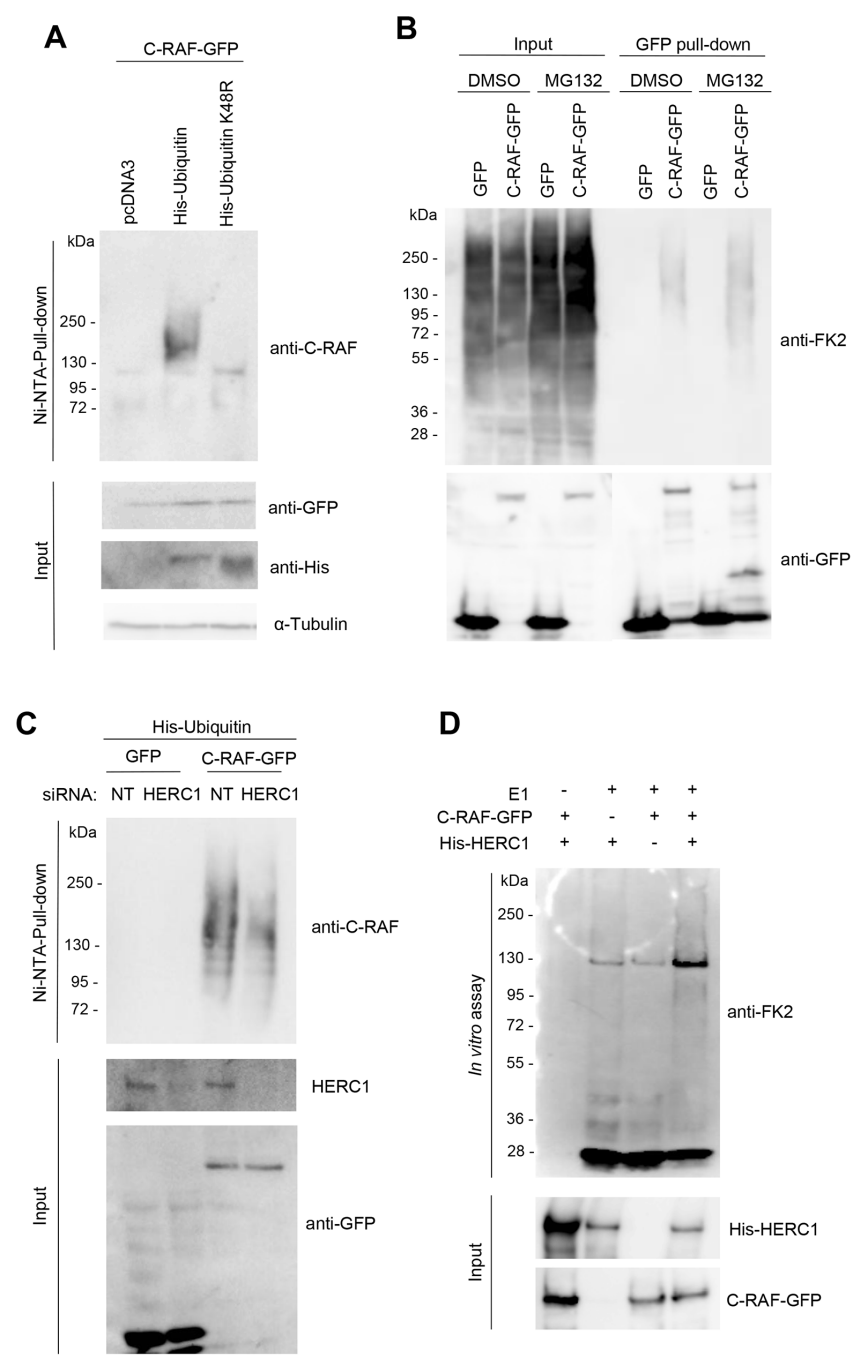
HERC1 regulates C-RAF ubiquitylation **(A)** HEK-293T cells were transfected with C-RAF-GFP and pcDNA3, His-Ubiquitin or His-Ubiquitin-K48R constructs. Twenty-four hours later, supernatants (input) were analyzed by immunoblotting with specific antibodies and His-tagged proteins purified using a Ni-NTA-agarose resin as indicated in “Materials and Methods”. The C-RAF protein retained in the resin was analyzed by immunoblotting with a specific anti-C-RAF antibody. **(B)** HEK-293T cells transfected with GFP or C-RAF-GFP were incubated in the presence of the proteasome inhibitor MG132 (10 μM) for 6 hours. Control cells were incubated with dimethyl sulfoxide (DMSO). GFP pull-down was performed as indicated in “Materials and Methods”. Proteins retained in the resin were analyzed by immunoblotting with the indicated antibodies. **(C)** HEK-293T cells were transfected with NT or HERC1 siRNAs. Twenty-four hours later, cells were transfected with His-Ubiquitin and GFP or C-RAF-GFP. Forty-eight hours later, His-tagged ubiquitin were purified using a Ni-NTA-agarose resin. Inputs and pull-downs were analyzed as in (A). **(D)** Purified His-HERC1 and C-RAF-GFP proteins were incubated in a buffer containing ATP, ubiquitin, and E1 and E2 enzymes as indicated in “Materials and Methods”. The reaction products were analyzed by immunoblotting. Data are representative of three independent experiments.

Previous reports have demonstrated ubiquitylation and degradation of C-RAF via the proteasome [37–40]. A polyubiquitin chain linked through lysine 48 is the principal signal for targeting substrates to the proteasome. To check whether C-RAF can be polyubiquitylated, HEK-293T cells were transfected with plasmids expressing C-RAF-GFP and His-Ubiquitin, or with the negative control pcDNA3. Twenty-four hours later, lysates from these cells containing His-Ubiquitin-tagged proteins were pulled-down using Ni-NTA agarose, a nickel-charged affinity resin for purifying recombinant proteins carrying a His tag. Inputs and purified proteins were analyzed by PAGE/SDS and immunoblotted with specific antibodies (Figure 10A). A characteristic polyubiquitylation smear was detected with the anti-C-RAF antibody (Figure 10A, lane 2). To show that the polyubiquitin chain was linked through lysine 48, a plasmid expressing His-Ubiquitin with lysine 48 mutated to arginine (K48R) was also transfected and processed as above. In these conditions, C-RAF polyubiquitylation was not observed (Figure 10A, lane 3). We have also analyzed C-RAF polyubiquitylation in the absence and presence of the proteasome inhibitor MG132 using the anti-ubiquitylated proteins (FK2) antibody. GFP pull-down of HEK-293T cells transfected with GFP or C-RAF-GFP showed an increase in polyubiquitylated C-RAF in the presence of MG132 (Figure 10B, lane 8 compared with lane 6).

Knowing that HERC1 is a member of the HECT superfamily of E3 ubiquitin ligases, that HERC1 knockdown increases the C-RAF level, and that C-RAF is polyubiquitylated through lysine 48, we wondered whether HERC1 could be regulating C-RAF ubiquitylation. To test this, HEK-293T cells were initially transfected with HERC1 or non-targeting siRNAs. Twenty-four hours later, cells were transfected with plasmids expressing His-Ubiquitin and C-RAF-GFP, or GFP as a negative control. Forty-eight hours later, lysates from the cells containing His-Ubiquitin-tagged proteins were pulled-down with Ni-NTA resin. Inputs and purified proteins were analyzed by PAGE/SDS and immunoblotting (Figure 10C). We observed that the polyubiquitylation smear detected with anti-C-RAF antibody was decreased after HERC1 knockdown. These data show that HERC1 regulates C-RAF ubiquitylation. To analyze whether C-RAF can be the ubiquitylation substrate of HERC1, we performed an *in vitro* ubiquitylation assay. When purified His-HERC1 and C-RAF-GFP proteins were incubated in a buffer that contained ATP, ubiquitin, and E1 and E2 enzymes, C-RAF polyubiquitylation was observed (Figure 10D).

## DISCUSSION

HERC1 is a giant protein that contains in its primary structure a HECT domain, which is characteristic of a large family of E3 ubiquitin ligases [9]. The present study reveals an important role of HERC1 in cell homeostasis. Our results show how dysregulation of HERC1 function by its interference RNA increases cell proliferation. These data are consistent with previous studies that would suggest an association between HERC1 mutations and cancer [25, 26]. We demonstrate how HERC1 knockdown regulates cell proliferation by means of activation of ERK signaling through a mechanism dependent on RAF activity. We show that HERC1 interacts with RAF proteins and specifically modulates C-RAF stability, regulating its polyubiquitylation. We conclude that the E3 ubiquitin ligase HERC1 controls cellular proliferation through activation of ERK signaling by regulating C-RAF protein levels via ubiquitylation.

These data implicate for the first time the E3 ubiquitin ligase HERC1 in the model of regulating C-RAF activity. It has been reported that C-RAF autophosphorylation at Serine 621 stabilizes C-RAF,inhibiting its degradation by the proteasome [40]. Interestingly, in the absence of Serine 621 phosphorylation, C-RAF is degraded by the proteasome by mechanisms that involves an unknown E3 ubiquitin ligase [40–41]. Our results are compatible with the participation of HERC1 in this model, where C-RAF is marked with a polyubiquitylation signal for degradation by the proteasome. The level of C-RAF protein would be determined by an equilibrium between the activity of E3 ubiquitin ligase of HERC1 (degradation) and its own kinase activity (stability). In this model, and in accordance with our observations, HERC1 depletion would increase the amount of C-RAF and this would be enough to activate the ERK signaling pathway.

The RAF/MEK/ERK signaling pathway regulates fundamental cellular processes including growth, proliferation, differentiation, survival and migration [1]. The importance of this regulation is revealed when the pathway is altered. Aberrant regulation of this signaling pathway has long been associated with human cancers. For this reason, it has been the subject of intense research and pharmaceutical scrutiny to identify target-based approaches for cancer treatment [3, 42]. Currently, inhibitors of RAF activity probably represent the most studied approach for blocking ERK signaling. For example, the inhibitor of RAF activity Sorafenib is used as a therapy in renal cell carcinoma, hepatocellular carcinoma, melanomas and multiple myeloma [3, 42–44]. This inhibitor led to a significant disruption of Serine 621 phosphorylation and decreased stability of C-RAF [40]. The antitumor activity of the antibiotic Geldanamycin and its analogues is due to binding to and promoting HSP90 degradation; HSP90 functions as a chaperone that is required for the stability and function of C-RAF. Hence, it indirectly inhibits the function of C-RAF by promoting its proteasomal degradation [3]. A similar effect would be observed stimulating the ubiquitin-ligase activity of HERC1, which would specifically promote the proteasomal degradation of C-RAF. In this context, it has been shown that C-RAF is essential for development of *K-RAS* oncogene-driven non-small cell lung carcinoma (NSCLC) [45]. The search and identification of pharmaceutical stimulators of HERC1 activity could have great therapeutic potential in a variety of tumor types, including NSCLC, which is driven by the *RAS* oncogene most frequently mutated in human cancer.

HERC1 was discovered more than 20 years ago [11]. During this time, several proteins such as ARF, Rab, Clathrin, M2-pyruvate kinase, TSC2 and BAK have been described to interact with HERC1 [9]. Now, we demonstrate that RAF proteins also form part of the HERC1 interactome. Although some of the above proteins may participate in processes regulated by HERC1, to our knowledge, C-RAF is the first substrate reported for the E3 ubiquitin ligase activity of HERC1. It is intriguing to observe that despite its interaction with all RAF isoforms, HERC1 specifically targets C-RAF for degradation. Interestingly, knockdown experiments (Figure 4A and Figure 5) and experiments with RAF inhibitors (Figure 4B-C) show the involvement of all RAF isoforms in the regulation of ERK signaling and cell proliferation by HERC1.

In summary, we identify a new function for the E3 ubiquitin ligase HERC1 as a regulator of ERK signaling. Our findings indicate that HERC1 interacts with RAF proteins and controls C-RAF stability through regulation of its polyubiquitylation. These data show the important role of HERC1 in cell homeostasis, contribute to clarify the activation model of C-RAF, and point out pathological consequences of HERC1 dysregulation.

## MATERIALS AND METHODS

### Reagents

Anti-p-ERK1/2 (Sigma-Aldrich); anti-HERC1 (Bvg6 and 410) [46]; anti-Clathrin heavy chain and anti-C-RAF (BD Biosciences); anti-B-RAF (F-7) and anti-A-RAF(A-5) (Santa Cruz Biotechnology); anti-p44/42 MAPK (ERK1/2) and anti-His-Tag (27E8) (Cell Signaling); anti-GFP (Abcam); anti-α-Tubulin (Calbiochem); anti-ubiquitinylated proteins (clone FK2) (Biomol); horseradish peroxidase-conjugated secondary antibodies, and Alexa-Fluor 488 and 555 conjugated secondary antibodies (Invitrogen); anti-chicken IgY-peroxidases (Sigma-Aldrich) protein A-Sepharose and protein G-Sepharose (GE Healthcare); Phalloidin-Alexa 647 (BioProbes); DAPI (Sigma-Aldrich); Immobilon-P PVDF transfer membrane (Millipore Corp.); GFP-TrapA (Chromotek); U0126 (Calbiochem); Sorafenib (Santa Cruz Biotech.); LY3009120 (Selleckchem); MG132 (Merck Millipore); Ni-NTA Agarose (Qiagen); Recombinant Human His_6_-Ubiquitin-activating Enzyme/UBE1 (Boston Biochem); EGF (PeproTech); Ubiquitin (human- recombinant) (Enzo).

### Plasmids and siRNAs

C-RAF-myc-His construct, C-RAF-GFP construct, His-Ubiquitin constructs (WT and K48R) and pT7-7-His-UbcH5 constructs (a, b and c) were provided by Dr. A. Bajljuls/M. Halasz [47], Dr. T. Balla [48], Dr.T. Erazo [49] and Dr. K. Iwai [50], respectively. GFP-HERC1 fusion constructs (1-412, 1-1334, 1-2958, 365-794, 754-1334, and 3152-461), and myc-HERC1 (1-1413) were generated from human HERC1 cDNA [11] by digestion with appropriated restriction enzymes and subcloning in pEGFP or pCMV-Tag3 vectors, respectively. GFP-C-RAF fusion constructs: cDNAs encoding residues 1–200, 201-300 and 301-648 of human C-RAF were amplified by PCR using specific oligonucleotides. Amplified fragments were digested with Eco RI/Sma I restriction enzymes and subcloned into pEGFP-C2. Plasmids were sequenced. Baculovirus encoding human His-HERC1 was previously reported [13]. MISSION shRNA clone of human HERC1 (TRCN0000235499) and the lentivirus plasmid vector pLKO.1-Puro were purchased from Sigma-Aldrich. The following siRNAs were used in this study: two siRNAs targeting the human sequence of HERC1 (Q1, CGGCAUGGAUGAACAAAUU and Q4, GGGCAGAACUUCGUUUAGA); non-targeting (NT) siRNA (NT, UAGCGACUAAACACAUCAA); A-RAF siRNA (AACAACATCTTCCTACATGAG); B-RAF siRNA (AAAGAATTGGATCTGGAT CAT); C-RAF siRNA (UAGUUCAGCAGUUUGGCUATT) were purchased from GenePharma.

### Cell culture and transfections

HeLa, HEK-293T, U2OS, H1299 and A549 cells were cultured at 37°C in Dulbecco’s Modified Eagle’s Medium (DMEM) (Gibco) with 10% fetal bovine serum. Transfection of cells was carried out using calcium phosphate for siRNAs, and polyethylenimine (PEI) or Lipofectamine LTX (Invitrogen) for plasmids. The final concentration of siRNAs was 100 nM and the plasmid DNA amount was 2 μg (for lipofectamine LTX) or 8 μg (for PEI). Cells transfected with plasmids were analyzed 24-48 hours later. Cells transfected with plasmids and siRNAs or only with siRNAs were recovered at 72 hours post-transfection. Specific inhibitors were used at a final concentration of 10 μM (U0126 for 1 hour, Sorafenib and LY3009120 for 2 hours, and MG132 for 6 hours). Preparation of lentiviral transduction particles and their infection were performed following the indications of the manufacturer Sigma-Aldrich.

### Lysates and immunoblotting

Cells or tissues were lysed with NP40 buffer (50 mM Tris-HCl, pH 7.5, 150 mM NaCl, 50 mM NaF, 0.5% NP40) containing protease and phosphatase inhibitors (50 mM β-glycerophosphate, 1 mM sodium vanadate, 1 mM phenylmethylsulfonyl fluoride, 5 μg/ml leupeptin, 5 μg/ml aprotinin, 1 μg/ml pepstatin A, 100 μg/ml benzamidine and 1μM E-64). Lysates were maintained on ice under agitation for 20 min, and then centrifuged at 13,000 g at 4°C for 10 min. Supernatants were collected to be analyzed using the Tris-Acetate PAGE system [51]. Band intensities were detected using a gel documentation system (LAS-3000, Fujifilm) and quantified with ImageJ software. Protein levels were normalized and expressed as a percentage of controls.

### Immunoprecipitation and pull-downs

For immunoprecipitation (IP), supernatants (input) were incubated with pre-immune serum (PI) or with anti-HERC1 polyclonal antibody (Bvg6) for 2 hours at 4°C with gentle rotation and immunoprecipitated with protein A-Sepharose for 1 hour at 4°C. Beads were pelleted by centrifugation at 2,500 g, washed four times with NP40 buffer, and analyzed by electrophoresis and immunoblot as it was indicated above. For GFP pull-downs, supernatants were incubated with 3μL of GFP-TrapA for 2 hours at 4°C. Pellets were washed four times with NP40 buffer and analyzed by electrophoresis and immunoblotting as indicated above.

### Clonogenic assay

For clonogenic assays, U2OS and HeLa cells were transfected with the indicated siRNAs and 24 hour post transfection cells were trypsinized and 1000 cells were reseeded in a DMEM with 5% new born calf serum and grown until cells formed sufficiently large colonies (12-15 days). The colonies were stained with crystal violet and quantified by absorbance at 550 nm.

### Confocal microscopy

HEK-293T cells were grown on glass coverslips and fixed with 4% paraformaldehyde for 20 min at room temperature. Cells were blocked and permeabilized with 0.5% bovine serum albumin and 0.05% Saponin in PBS for 20 minutes. The primary antibodies, anti-myc (1:100), anti-C-RAF (1:100) and anti-HERC1 (410; 1:100), were incubated at 37°C for 1 hour. After washing, secondary antibodies (1:500) were incubated at 37°C for 45 minutes. F-actin was detected incubating for 20 minutes at room temperature with Phalloidin-Alexa 647 (100 ng/ml). Nuclei were stained with DAPI (1 μg/ml). Images from optical sections (thickness: 0,31 μm) were acquired using a Carl Zeiss LSM 880 spectral confocal laser scanning microscope (Carl Zeiss Microscopy GmbH, Jena, Germany) using a 63x oil immersion objective (1.4 numerical aperture) and image resolution of 1024 X 1024 pixels. Pearson’s correlation coefficient (POC) and Manders’ overlap coefficient (MOC) were calculated using ImageJ software.

### Protein cross-linking assay

Cells were transfected with the indicated siRNAs and lysed in NP40 buffer 72 hours after transfection. After lysis, cells were centrifuged, and the supernatant was recovered. Glutaraldehyde was added to the supernatant at the indicated concentrations and incubated on ice for 30 min, as described previously. The reaction was stopped with sample buffer (1x final concentration) and samples were analyzed by immunoblotting analysis as indicated above.

### Ubiquitylation *in vivo* and *in vitro* assay

For the *in vivo* ubiquitylation assay, we followed the method described by Erazo et al. [49]. In brief, HEK-293T cells were transfected with the indicated siRNAs and plasmids for 72 hours and harvested by denaturing buffer (6 M guanidinium-HCl, 10 mM Tris, 100 mM Na_2_HPO_4_-NaH_2_PO_4_ buffer, pH 8). Cells extracts were then incubated with the nickel beads (Ni^2+^-NTA) for 2 hours at 4°C with rotation. Beads were successively washed as follows: twice with 1 ml of buffer 1 plus 10 mM 2-mercaptoethanol; three times with 1 ml of buffer 2 (8 M urea, 10 mM Tris, 10 mM 2-mercaptoethanol, 100 mM Na_2_HPO_4_- NaH_2_PO_4_ buffer, pH 8); twice with 1 ml of buffer 3 (8 M urea, 10 mM Tris, 100 mM Na_2_HPO_4_-NaH_2_PO_4_ buffer, pH 6.3) containing 0.2% Triton X-100; once with 1 ml of buffer 3 containing 0.1% Triton X-100 and 0.5 M NaCl; and three times with 1 ml of buffer 3. Finally, proteins were eluted by incubating the beads with 200 mM imidazole in 5% SDS, 0.15 M Tris-HCl, pH 6.7, 30% (vol/vol) glycerol, 0.72 M 2-mercaptoethanol for 1 hour at 37°C with mixing. The samples were analyzed by immunoblotting analysis as indicated above.

For the *in vitro* ubiquitylation assay, His-HERC1 protein was purified from Sf9 cells infected with HERC1 baculovirus using Ni-NTA resin [13]. C-RAF-GFP was purified from HEK-293T using GFP-TrapA beads. E2 enzymes were purified from bacteria transformed with plasmids expressing His-UbcH5a, His-UbcH5b and His-UbcH5c proteins [13, 50]. Commercial purified E1 enzyme and ubiquitin were also used. Purified HERC1 and C-RAF-GFP were incubated for 2 hours at 30°C in 50μL reaction buffer containing 0.5μg E1, 2μM ubiquitin, 1μg His-UbcH5a, 1μg His-UbcH5b, 1μg His-UbcH5c, 2.5mM ATP, 5mM MgCl_2_, 5mM KCl, 1mM DTT and 20mM HEPES (pH7.3). The reaction products were analyzed by immunoblotting analysis as indicated above.

### Statistical analysis

Data are presented as mean ± SEM. Statistical significance was determined by Student’s *t* test or One-way Anova using GraphPad Prism 5 software. p values under 0.05 were considered statistically significant.
